# Lessons From an Early-stage Epidemiological Study of Minamata Disease

**DOI:** 10.2188/jea.JE20190089

**Published:** 2020-01-05

**Authors:** Takashi Yorifuji

**Affiliations:** Department of Epidemiology, Graduate School of Medicine, Dentistry and Pharmaceutical Sciences, Okayama University, Okayama, Japan

**Keywords:** environment and public health, epidemiology, food contamination, methylmercury compounds, Minamata disease

## INTRODUCTION

The Revisit series in this issue introduced the article by Kitamura and colleagues.^1^ Dr. Shoji Kitamura, born in 1915, was a medical doctor and a professor of Department of Public Health in the Medical School at Kumamoto University when Minamata disease happened. The article summarized findings from a very-early-phase epidemiological study conducted by researchers from Kumamoto University immediately after the Minamata disease incident was officially recognized on May 1, 1956. The epidemiological study was very well-conducted in a timely manner and the article was available as early as January 1957 in an academic journal published by the Medical School at Kumamoto University. This is a very influential report that demonstrated associations between fish intake and the Minamata disease after careful descriptive and analytical epidemiological studies. Although the Japanese society should have taken some actions to prevent the disease with the evidence that a research group at Kumamoto University had at that time, the pollution was not stopped until 12 years had passed since the official identification in 1956. Moreover, the struggle with Minamata disease is still in progress. This unfavorable response by the Japanese society could partly be explained by the important economic role of the causative factory at that time, when Japan had recorded a trade deficit since the end of the Second World War.^2^ Plastic products of the factory were key Japanese exports helping to reduce this deficit. In this commentary, after introducing the study by Kitamura and colleagues, I discuss the potentials of epidemiology, as well as consequences on public health that occurred when we did not follow the findings from the epidemiological study.

## MINAMATA DISEASE

Minamata disease is a large-scale methylmercury food poisoning that occurred in Minamata and neighboring communities in Japan during the 1950s and 1960s.^2^^,^^3^ Affected patients manifested neurological signs, including sensory disturbance, ataxia, dysarthria, constriction of the visual field, and hearing difficulties.^4^ Up to January 2019, 2,282 patients have been officially recognized as having Minamata disease in Kumamoto and Kagoshima Prefectures,^5^ but it is reported that several tens of thousands of residents have neurological signs related with methylmercury poisoning in the exposed area.^2^^,^^6^ The causative factory, located in Minamata City, released effluent, which included methylmercury as a byproduct of acetaldehyde production and contaminated local seafood. The acetaldehyde production started in 1932, and it increased after the World War II, with a peak in 1960, and stopped in 1968. Along with the increase in production from around 1950, local residents witnessed strange phenomena.^7^ For example, large number of fish rose to the surface and swam crazy, sea birds became unable to fly, and local cats exhibited strange behavior, such as drooling and running in circle as though they were mad. Finally, two young sisters aged 2 and 5 years showing neurological disorders with unknown causes were officially notified to the local public health center on May 1, 1956. This was the official identification of Minamata disease and the beginning of it.

## AN EPIDEMIOLOGICAL STUDY BY KITAMURA AND COLLEAGUES

After the official identification, local doctors identified numerous new cases having neurological signs with unknown causes, and 34 cases, including 13 deaths, were identified by August 1956.^8^ In response to a request from the local doctors, a local university (Kumamoto University) established a Research Group that included various medical departments in August 1956. In the epidemiological section, Shoji Kitamura and his group conducted both a descriptive and an analytic epidemiological study. Focusing on 40 households with patients and 68 adjacent households without patients, they performed the study in a very detailed manner, taking into account various potential exposures, such as local geographical and meteorological conditions, livestock, drinking water, and foods.

In the descriptive study, following the principle of descriptive epidemiology (ie, time, place, and person),^9^ they examined the time trend of patients correlating it with amount of fishing in the exposed area, plotted the locations of the patients, and examined the characteristics of the patients in a detailed manner. In particular, they plotted the time sequence of cases on a map (Figure 4 in the article^1^) and speculated that the disease was not contagious, which is a very remarkable discovery in the early phase of the incident.

Moreover, they examined an association between family occupation of the households and the disease in the analytical epidemiological study (Table 8 in the article^1^) and demonstrated that the households with the patients had higher odds of fishing occupation compared with the control households (odds ratio 21.6; 95% confidence interval, 6.8–68.7). They further focused on intake of fish caught in Minamata Bay (which is the bay where the effluent from the factory was discharged into) and demonstrated that the households with the patients had higher odds of eating fish caught in the bay compared with the control households (Table 17 in the article^1^). For example, odds ratio of eating fish caught in the bay almost every day was 26.7 (95% confidence interval, 8.1–88.2). Because it seems that there is no confounding factor, such crude analyses should have provided valid results.

Finally, Kitamura and colleagues concluded that the disease could be induced by continuous exposure to a common factor, which seemed to be contaminated fish in Minamata Bay. They also raised several potential sources of pollution that contaminated the fish in the end of the article and the causative factory was listed at the top.

## RESPONSE

Based on the findings from various medical departments, including that from the epidemiological study, the Research Group of Kumamoto University reported that the disease was not contagious but a food poisoning incident by intake of contaminated fish from the Minamata Bay, and it was caused by heavy metal, probably from the factory’s effluent in November 1956. Moreover, the scientific research team of the Ministry of Health and Welfare of Japan also started an epidemiological investigation in Minamata.^10^ In March 1957, they demonstrated a positive association between family occupation (fishing) and the disease, consistent with the epidemiological study by Kitamura and colleagues. Then, they concluded that the disease could be induced by contaminated fish in Minamata Bay and the factory and its effluent should be fully investigated to elucidate the disease’s mechanism.

In response to these findings, the local prefecture (Kumamoto Prefecture) considered the use of the Food Sanitation Act in March 1957 to regulate the consumption of contaminated fish and asked for the opinion of the Japanese Government.^11^ Subsequently, on September 11^th^, 1957, the Ministry of Health and Welfare of Japan replied to the local Government that it was impossible to apply the Food Sanitation Act because there was no clear evidence that “all” fish and “all” shellfish are poisoned in the specified area in Minamata Bay.^12^ As a result, the residents continued to eat contaminated fish without any effective measure, and no further epidemiological investigation, which should be conducted by a local public health center based on the Act, was performed.

## AFTER THE FAILURE OF RESPONSE

After the epidemiological finding did not alter the attitudes of the local prefecture and the Japanese government, epidemiological studies disappeared from the front stage of Minamata disease incident, and researchers devoted themselves to laboratory studies.^7^ Because there was no appropriate measure to control the outbreak, the researchers at Kumamoto University made efforts to find the etiological agent(s) of the disease and found that the etiological agent was methylmercury in 1959. However, there were no steps taken to control the poisoning.

Therefore, researchers were eager to find the mechanism by which methylmercury was produced. They succeeded in 1962, when methylmercury chloride was extracted from the sludge of an acetaldehyde production process in the causative factory and it was demonstrated that methylmercury was produced as a byproduct in the process of producing acetaldehyde. However, no regulation of the factory was conducted.

Subsequently, in January 1965, similar methylmercury food poisoning occurred in Niigata, the so-called Niigata Minamata disease and the factory that was responsible for the disease operated in the same way as the factory in Minamata. After the case relating to Niigata Minamata disease went on trial in 1967, the Japanese government officially acknowledged the causal relationship between wastewater from the factory in Minamata and Minamata disease in September 1968. However, methylmercury production had already stopped by May 1968, since it had already become unnecessary for the factory to produce acetaldehyde. Twelve years had passed since the causal food was identified. During the period, the residents continued to eat contaminated fish without any effective preventive measure, and the exposure spread not only in Minamata Bay but also along the entire coast of the Shiranui Sea (a large inland sea that Minamata Bay is connected with).

After the Japanese government accepted the causal relationship between the factory and Minamata disease in 1968, attention shifted to the accreditation and compensation for the patients. Interested readers can read the following references for more detailed information on the history of Minamata disease.^2^^,^^7^^,^^11^^,^^13^ One point which should be stressed is that, if the appropriate response was conducted following the findings from the epidemiological study by Kitamura and colleagues, the damage to humans, animals, and ecosystems would have been much smaller.

## IMPLICATION

In this commentary, I briefly introduced the history of Minamata disease, with an overview on the epidemiological study by Kitamura and colleagues. The Minamata disease incident provides a lot of lessons on epidemiology and public health,^2^ but the Kitamura article and the subsequent response illuminates the potential of epidemiology, as well as consequences on public health that occurred when we did not follow the findings from the epidemiological study. The failure of response not only expanded the exposure and increased the number of affected residents, but also obscured the epidemiological features of the disease (such as the threshold, frequency of signs, and the scale of poisoning) because the researchers at Kumamoto University devoted themselves to laboratory studies and epidemiological studies disappeared from the front stage.

The history of Minamata disease provides many examples of inappropriate burdens of proof, which prevented speedy and effective action. The demand for high levels of scientific proof (ie, “all fish and all shellfish are poisoned”, “etiologic agent”, or “mechanism”) was used to delay the regulation of methylmercury pollution. Epidemiology demonstrated that poisoning was caused by contaminated fish in the Minamata Bay, and the researchers suspected the factory discharge already in 1956. As Goodman et al described, whether investigation or control has priority depends on the levels of certainty about the etiology and source/mode of transmission.^14^ When we look back over the history of Minamata disease, the source/mode of transmission (ie, eating contaminated seafood) was proven in 1956, but the etiological agent(s) were not fully demonstrated. Therefore, both investigation and control should have been conducted, although no effective control was undertaken and the residents continued to be exposed. This example tells us an important lesson: “Prompt countermeasures should be conducted when a cause is identified and should not be postponed until an etiological agent or mechanism is identified.”^2^

One point should be added to an interpretation on the Kitamura’s article. In the article, they concluded that “there has been no infant case”, but it was not true. At that time, many children were born with conditions resembling cerebral palsy in the exposed area,^15^ later they were known as congenital Minamata disease patients who were affected by methylmercury in utero during the exposure period. However, it took a long time for congenital Minamata disease patients to be accepted as a truth because it was believed that the placenta could protect the fetuses from foreign substances at that time. Ultimately, in December 1962, 17 children with symptoms resembling cerebral palsy were officially diagnosed with congenital Minamata disease patients.

In conclusion, the epidemiological study by Kitamura and colleagues is historical but one of the most valuable epidemiological studies conducted in Japan. Their conclusion that eating fish caught in Minamata Bay was the source of the disease has never changed, which was determined 3 years before the etiologic agent was found and six years before the mechanism was discovered. Kitamura’s article demonstrates the potential of epidemiology and the consequences on public health when we did not follow the epidemiological findings. Early epidemiological studies can play a key role in preventing and minimizing future harm.
